# Supplementary material for: Polyketide synthase gene clusters in the ericoid mycorrhizal fungus Oidiodendron maius: genome-wide prediction, expression profiling, and functional characterization of knockout mutants
Source: Front Plant Sci. 2026 Sep 16;17:1924430. doi: 10.3389/fpls.2026.1924430 (PMC13623903; doi:10.3389/fpls.2026.1924430)
Supplement: Supplementary file 1 [file SupplementaryFile1.zip › Supplementary Materials/Supplementary Folder 1.zip!/Oidma1_AssemblyScaffolds_Repeatmasked/knownclusterblast/cluster44/jgi.p_Oidma1_74151_mibig_hits.html]

| MIBiG Protein | Description | MIBiG Cluster | MiBiG Product | % ID | % Coverage | BLAST Score | E-value |
| --- | --- | --- | --- | --- | --- | --- | --- |
| AHG26149.1 | FAD-linked\_oxidoreductase\_YvdP-like\_protein | BGC0000812 | Huperzine\_A\_biosynthetic\_gene\_cluster | 46 | 100.0 | 396 | 4.1e-110 |
| EAQ86396.1 | hypothetical\_protein | BGC0001405 | Chaetoviridin\_/\_chaetomugilin\_biosynthetic\_gene\_cluster | 37 | 99.7757847534 | 280 | 4.3e-75 |
| CAP95408.1 | unnamed | BGC0001404 | Sorbicillin\_biosynthetic\_gene\_cluster | 36 | 99.7757847534 | 265 | 1.4e-70 |
| AAN85480.1 | putative\_FAD-dependent\_oxygenase | BGC0001101 | Leinamycin\_biosynthetic\_gene\_cluster | 28 | 100.67264574 | 128 | 2.7e-29 |
| AHA81986.1 | Arm16 | BGC0000199 | Arimetamycin\_biosynthetic\_gene\_cluster | 26 | 105.605381166 | 121 | 3.3e-27 |
| ABI22129.1 | oxidoreductase | BGC0000422 | Saframycin\_A\_biosynthetic\_gene\_cluster | 35 | 48.8789237668 | 113 | 9e-25 |
| ABI22120.1 | oxidoreductase | BGC0000422 | Saframycin\_A\_biosynthetic\_gene\_cluster | 33 | 44.8430493274 | 104 | 3.2e-22 |
| CAJ42334.1 | dehydrogenase | BGC0000273 | Steffimycin\_biosynthetic\_gene\_cluster | 31 | 28.4753363229 | 65 | 2.2e-10 |
| AHB38495.1 | EpxB | BGC0000346 | Epoxomicin\_biosynthetic\_gene\_cluster | 32 | 26.9058295964 | 61 | 3.1e-09 |
| AKA59434.1 | FAD-linked\_oxidase | BGC0001202 | Landepoxcin\_biosynthetic\_gene\_cluster | 31 | 32.2869955157 | 61 | 4.1e-09 |
| CCC55910.1 | putative\_dehydrogenase | BGC0000973 | Collismycin\_A\_biosynthetic\_gene\_cluster | 27 | 27.5784753363 | 60 | 6.9e-09 |
